# Human Heart Morphogenesis: A New Vision Based on In Vivo Labeling and Cell Tracking

**DOI:** 10.3390/life13010165

**Published:** 2023-01-06

**Authors:** Laura Villavicencio-Guzmán, Concepción Sánchez-Gómez, Ricardo Jaime-Cruz, Tania Cristina Ramírez-Fuentes, Carlos César Patiño-Morales, Marcela Salazar-García

**Affiliations:** 1 Research Laboratory of Developmental Biology and Experimental Teratogenesis, Children’s Hospital of México Federico Gomez, Mexico City 06720, Mexico; 2 Departamento de Ciencias de la Salud, Universidad Tecnológica de México—UNITEC México—Campus Sur, Mexico City 09810, Mexico; 3 Sección de Estudios de Posgrado e Investigación, Escuela Superior de Medicina del Instituto Politécnico Nacional, Mexico City 11340, Mexico; 4 Laboratorio de Biología Celular, Universidad Autónoma Metropolitana-Cuajimalpa, Mexico City 05348, Mexico; 5 Facultad de Medicina, Universidad Nacional Autónoma de México, Mexico City 04360, Mexico

**Keywords:** cardiogenesis, heart morphogenesis, cell tracking, embryo

## Abstract

Despite the extensive information available on the different genetic, epigenetic, and molecular features of cardiogenesis, the origin of congenital heart defects remains unknown. Most genetic and molecular studies have been conducted outside the context of the progressive anatomical and histological changes in the embryonic heart, which is one of the reasons for the limited knowledge of the origins of congenital heart diseases. We integrated the findings of descriptive studies on human embryos and experimental studies on chick, rat, and mouse embryos. This research is based on the new dynamic concept of heart development and the existence of two heart fields. The first field corresponds to the straight heart tube, into which splanchnic mesodermal cells from the second heart field are gradually recruited. The overall aim was to create a new vision for the analysis, diagnosis, and regionalized classification of congenital defects of the heart and great arteries. In addition to highlighting the importance of genetic factors in the development of congenital heart disease, this study provides new insights into the composition of the straight heart tube, the processes of twisting and folding, and the fate of the conus in the development of the right ventricle and its outflow tract. The new vision, based on in vivo labeling and cell tracking and enhanced by models such as gastruloids and organoids, has contributed to a better understanding of important errors in cardiac morphogenesis, which may lead to several congenital heart diseases.

## 1. Introduction

The new understanding of normal cardiac embryology is fundamental for the analysis, diagnosis, and regionalized classification of congenital heart defects (CHDs), which usually manifest at birth. CHDs affect nearly 1% of live newborns, with a higher incidence in preterm births and stillbirths [1]. The severity of CHDs is determined by the stage of gestation during which development is disturbed, the extent of impairment, and the embryo’s ability to compensate for the defect [2]. CHDs can be caused by defects in embryogenesis between the 3rd and 8th weeks of gestation when the main structures of the cardiovascular system develop. However, the origin of CHDs remains unknown. Genetic factors are observed in approximately 13% of cases, including the specific loci involved in familial cases of CHD and certain chromosomal abnormalities such as trisomies 13, 15, 18, and 21. Currently, the most widely known genetic defects are autosomal dominant mutations that cause the loss or gain of function of a particular protein, predominantly transcription factors. In the mid-1980s, a study conducted in the United States reported that approximately 25% of children with CHDs had associated extracardiac malformations, and approximately one-third of them had a known underlying genetic syndrome, including chromosomal abnormalities and Mendelian disorders, or an unknown syndrome, thereby highlighting the importance of genetic factors in the pathogenesis of CHDs [3]. More recent studies have confirmed these findings, emphasizing the prevalence of extracardiac abnormalities in patients with severe forms of CHD. In addition, skeletal, gastrointestinal, and genitourinary abnormalities, as well as neurodevelopmental disorders, are the most commonly associated features [4,5].

In 87% of CHD cases, the most widely accepted etiologies are environmental exposure, including congenital rubella syndrome, teratogens, diabetes during pregnancy, and other maternal factors such as obesity and lifestyle. In the field of the developmental origins of health and disease (DoHaD), it has been reported that epigenetic changes triggered by adverse environmental factors directly influence the phenotype and translate into pathological conditions. Thus, epigenetic processes, such as DNA methylation and histone modifications, are involved in the development of the heart [6].

Changes in the environment and maternal factors in the early stages of pregnancy may cause subtle changes in the activity of certain transcription factors, which may resemble the defects caused by hereditary mutations [7]. For example, GATA 4, NKX2-5, MEF2, and HAND2, as well as epigenetic regulators such as p300, SMYD1, EZH2, and BRG1, play critical roles in both health and disease processes [8,9]. It has been demonstrated that many of the histone-modifying enzymes that regulate remodeling in disease processes also play a role in normal heart development. This indicates the existence of a parallelism between the mechanisms that direct normal development and the mechanisms that direct the development of congenital heart disease [10].

Understanding the etiology of CHDs requires an in-depth knowledge of not only the morphological changes that occur during the transformation of the primitive straight heart tube into a mature four-chambered organ with valves and two separate circuits but also the cellular mechanisms involved, the signaling pathways that govern them, and the genes involved.

Recent information on the diverse genetic and molecular features of cardiac development has not led to a better understanding of the origin of CHDs [11]. We believe this is because most genetic and molecular studies have been conducted outside of the context of the progressive anatomical and histological changes in the embryonic heart. Our aim was to analyze the key events of heart development from its origin, during the processes of twisting and folding, and until the phase prior to the start of septation. We integrated the findings of descriptive studies on human embryos and information from experimental studies on chick, rat, and mouse models, which have anatomical and embryonic characteristics similar to those of humans [12,13,14]. In addition, the main molecular aspects were discussed. The events have been described by indicating the approximate number of days of human gestation according to the chronology of Carnegie and Streeter (1942) [15].

The review does not intend to list the numerous CHDs but rather to discuss new contributions to the knowledge of the normal development of different heart structures based on in vivo labeling studies, cell tracking, and breakthrough technology to create a new vision for the analysis and regionalized classification of CHDs.

## 2. Chronology of Heart Development

**Figure 1:**
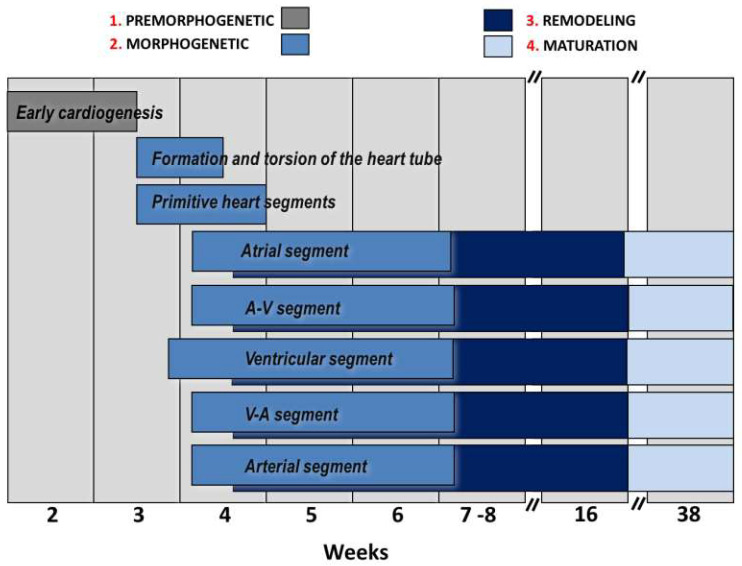
Chronology of the development of the human heart and the stages of the process. Abbreviations: A-V: atrioventricular, V-A: ventriculoatrial.

Heart development is a dynamic, sequential, morphologically ordered, and genetically and epigenetically regulated process that is usually divided into four stages that overlap in time (Figure 1).

1. Early cardiogenesis: It occurs during the premorphogenetic or presomitic stage of the embryo (days 8–18 of development). Early cardiogenesis begins with the organization of the cardiac areas and crescent through gastrulation and ends with the formation of two endocardial tubes that are externally covered by myocardial lineage cells.
2. Morphogenetic stage: This stage occurs during weeks 4–8 of embryonic development. It begins with the formation of the straight heart tube, derived from the first heart field (FHF), and ends after the integration of the primordia of all the structures that comprise the four-chambered heart, derived from the second heart field (SHF).
3. Septation and remodeling of the heart chambers: This stage begins during mid-embryonic development (day 30). At this stage, the primordia undergo differential growth and remodeling processes. The valves and septum are formed, and concurrently, the atrial and ventricular cavities acquire their morphological identities.
4. Maturation and histodifferentiation: It occurs during the fetal period (weeks 16–38) and involves histological maturation of the ventricular and atrial myocardium and histological differentiation of the ventriculoarterial and atrioventricular valve systems, including the tendinous cords and papillary muscles. Concurrently, the conduction system and coronary vessels are developed. Table 1 summarizes the cell lineages derived from both cardiac fields, the structures formed, and some congenital heart diseases that can occur during the abnormal development of said cardiac structures.

## 3. Early Cardiogenesis or Premorphogenetic Stage

**Figure 2:**
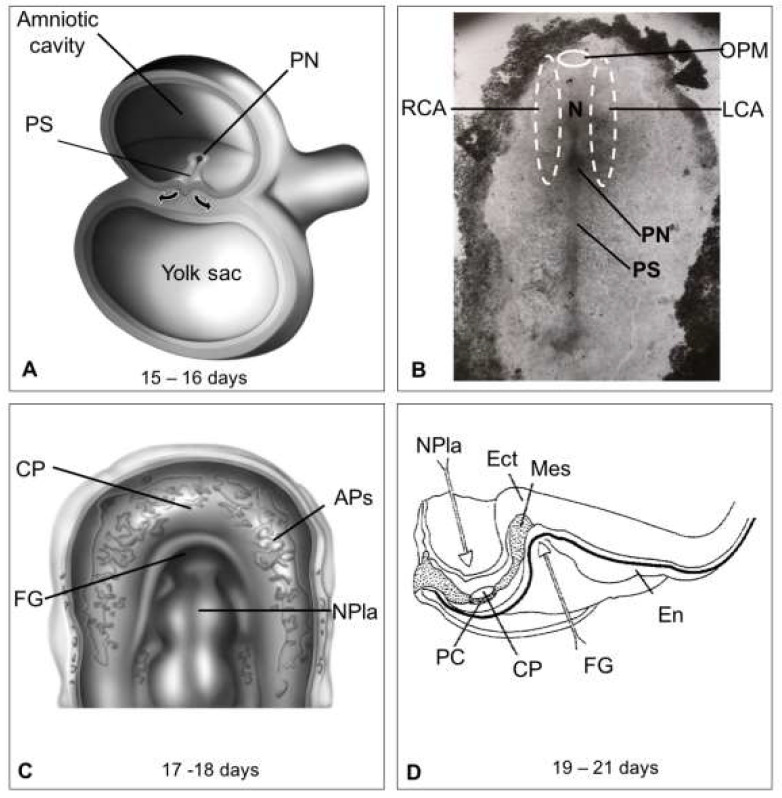
Diagrams and images of embryos at the gastrula stage. (**A**) Development of the trilaminar embryonic disk of chicken due to the migration (arrows) of epiblast cells through the primitive streak (PS). (**B**) A 16 ± 1-day embryo exhibiting the right (RCA) and left (LCA) cardiogenic areas spanning one-third of the primitive streak, the primitive node (PN), and the notochord (N) to the oropharyngeal membrane (OPM). (**C**) An 18 ± 1-day embryo showing the angiogenic plexuses (APs) arranged in the cardiac crescent, facing the neural plate (NPla). (**D**) Sagittal section of a 19–21-day human embryo with the first pair of somites showing the beginning of the development of the foregut (FG) and the neural plate. The somatopleure (SP), pericardial cavity (PC), and cardiogenic plate (CP) on the endoderm (En) of the yolk sac are also visible.

**Figure 3:**
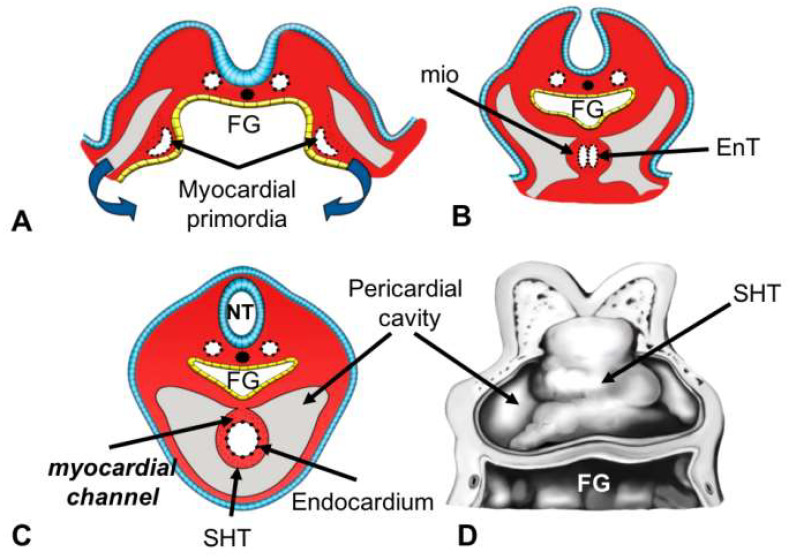
Formation of the heart in a straight tube (**A**–**C**). Cross-sectional diagrams of embryos depicting the tubulation process in embryos, which results in the fusion of the cardiac primordia to form the straight heart tube during late gastrulation (day 18 ± 1). (**A**) Note the two cardiac primordia composed of one endocardial tube and myocardial lineage cells (**B**), (**C**) Fusion of the two endocardial tubes (EnT) and formation of the straight heart tube (SHT), composed of one endocardial tube and a dorsally opened myocardial canal. (**D**) Frontal or ventral view of the straight heart tube derived from the cell populations of the first heart field. Abbreviations: FG: foregut, NT: neural tube.

The first cells with myocardial potential in the human embryo are identified at Carnegie Stage 7 (CS-7, days 15–16), i.e., when the embryo is a trilaminar disc (Figure 2A). At this stage, the heart precursors are located in two symmetrical areas of the mesoderm, known as cardiogenic areas, which are found on both sides of the primitive node and the notochord and reach the oropharyngeal membrane, the future site of mouth development (Figure 2B). During the late gastrula stage (CS-8, days 17–18), the endoderm forms the subcephalic pocket (precursor of the foregut). Simultaneously, populations of cardiac progenitor cells fuse at the midline cranial to the oropharyngeal membrane, forming a cardiogenic plate structure surrounding the incipient foregut. This structure is known as the cardiogenic plate or cardiac crescent (Figure 2C) and derives from the FHF [21,22]. Almost immediately (CS-9, days 19–21), the lateral plate mesoderm is separated by the intraembryonic coelom into the somatopleure, which is close to the ectoderm, and the splanchnopleure, which is close to the endoderm. The precursors of the cardiogenic plate are found in the splanchnopleure (Figure 2C,D) [23,24]. During this stage, the trilaminar embryo begins to bend cranially and cervically. The cardiac crescent is positioned ventral to the gut and forms angiogenic plexuses (Figure 2C,D). Following cavitation and remodeling, the latter forms two endocardial tubes covered by the myocardial mantle (day 22 ± 1), which are known as cardiac primordia because they are precursors of the endocardium and myocardium (Figure 3A). Shortly thereafter, the lateral margins of the embryo begin to fold at the sides of the yolk sac, initiating tubulation. As this process progresses, the endocardial tubes and their corresponding myocardial primordia move ventromedially (Figure 3B) until they merge and form a single myocardial tube, also known as the primitive heart tube or straight heart tube (Figure 3C,D) [24,25].

The differentiation of cardiomyocytes derived from the cardiac crescent depends on the signals from the adjacent endoderm. The endoderm and ectoderm express, in a complementary manner, bone morphogenetic protein (BMP) agonists and antagonists, fibroblast growth factor, and WNT, creating a unique signaling environment on the endodermal side that drives cardiac differentiation [26]. Thus, the cardiac crescent can be identified molecularly by the expression of several cardiogenic transcription factors and some sarcomere protein genes [23]. Concurrently, on the dorsal surface of the cardiac crescent, cells with cardiogenic capacity are recruited, thereby creating the SHF [27].

## 4. Morphogenetic Stage

Although most of the adult heart is derived from the splanchnic mesoderm, cardiac neural crest cells derived from the ectoderm contribute to some regions of the embryonic heart, such as the aortic sac and ridges of the embryonic outflow tract (Table 1). Mesothelium-derived cells, which form the epicardium, are also involved. The straight heart tube, derived from the FHF, initially consists of a closed endocardial tube and the dorsally open channel-shaped myocardial mantle (Figure 3C,D). The myocardial channel closes when the heart tube begins to twist and fold due to the bidirectional movement of the lateral edges of the myocardial mantle toward the dorsal midline [28]. Simultaneously, cardiac jelly, a term coined by Davis in 1927 [29], is deposited between the myocardium and endocardium [30]. Later, the cardiac jelly is redistributed to the regions where the mesenchymal tissue of the atrioventricular canal cushions and the embryonic outflow tracts will form. Subsequently, the epicardium is formed by the migration of precursor cells of mesothelial origin from the proepicardial organ, which are initially located on the pericardial side of the septum transversum and are also precursors of the coronary vessels [31]. The closure of the myocardial channel coincides with the first manifestation of contractile activity in the heart (days 21–22), which is essential for blood flow and cardiogenesis progression [32,33]. Many textbooks describe the straight heart tube as a segmented structure that already contains all of the components of the adult heart and only needs to grow [29]. It has also been proposed that it only consists of the primordia of the trabeculated regions of both ventricles [34]. Several studies published earlier this century reported that the heart grows by gradually adding cells at both ends of the heart tube to form the embryonic outflow tract, atria, and right ventricle [35,36,37,38]. This new concept revolutionized the field of heart development, although similar results were obtained from lineage tracing experiments in chick embryos in the 1970s [39].

**Figure 4:**
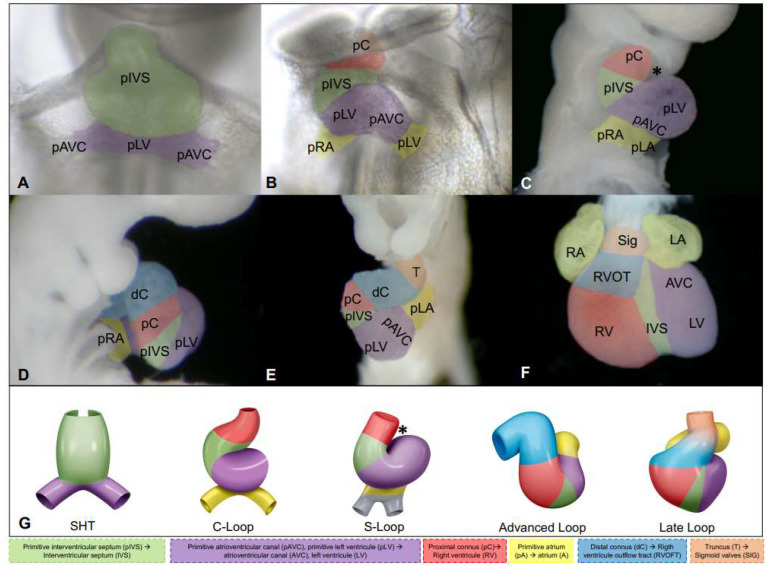
New proposal for the segmental pattern of the heart tube during twisting and looping. (**A**) Straight heart tube (SHT) and the cephalic region of SHT (green), which was previously considered the primordium of the right ventricle, participate in the development of the middle region and apical third of the IVS. The caudal region of the SHT (purple), the supposed primordium of the LV, also contributes to the development of the LV and AVC and is the last precursor of the inflow tracts of both ventricles and the outflow tract of the LV. At this stage, both primordial segments, pIVS and pLV, are connected in series: one cephalad and the other caudal. (**B**) Subsequently, the cardiac tube presents different conformational changes during the segmental incorporation of the primordia from the second cardiac field, C-loop. The proximal segment of the conus (brown), which actually participates in the development of the RV, is found in the C-loop. The right and left atrial primordia are caudally integrated. At this stage, the straight heart tube is turned to the right, and the ventral wall becomes part of the greater curvature of the loop. (**C**) S-loop. The elongation of the heart tube continues, and the lesser curvature of the loop (*) becomes more pronounced, causing both ends of the tube (inlet and outlet) to be closer to each other. (**D**) Advanced loop. The distal segment of the conus is present (blue) and forms the RVOFT. The anatomical position reached by each of the primordial cardiac segments stands out: the pLV is caudal to the PA and to the right of the RV. The DC and pRV descend and assume their correct anatomic positions. (**E**) Late loop (start of septation). The segment known as truncus (T) appears (pale pink) and forms the sigmoid arteries and their insertion annulus. (**F**) Mature heart. The heart is made up of four cavities that are properly connected and segmented: the right (RA) and left (LA) atriums, as well as the RV and LV. (**G**) Diagram summarizing the integration of the cardiac primordia during the torsion and folding stages of the cardiac tube. Abbreviations: PLA: primitive left atrium, PRA: primitive right atrium, Sig: sigmoid valves.

Based on molecular and cell tracking studies with fluorescent labeling in chick embryos and murine cell lines, it has been concluded that the straight heart tube is formed from the FHF. Nkx2.5 [40] and Tbx5 are among the molecular markers of the FHF [41]. In addition, in both birds and mammals, it has been shown that this primitive heart consists solely of cell populations that contribute to the development of left ventricular structures, cell populations that form the interventricular septum, and some atrioventricular populations (Figure 4A–F,G) [16,42]. The precursor regions of the remaining structures of the four-chambered heart are formed at later stages from cells of the SHF that are gradually recruited to the cranial and caudal ends of the developing heart tube (Figure 4B–F,G) [16]. The cells that form the straight heart tube do not proliferate. Therefore, the heart initially grows owing to the recruitment of cardiomyocytes derived from the SHF, which are located at the venous and arterial poles as well as in the dorsal mesocardium of the straight heart tube and whose molecular marker is the transcription factor ISL1 [38,42,43]. Following the rupture of the dorsal mesocardium, at the stage equivalent to CS-10 (days 21–22), the cells can only be recruited at the venous and arterial poles of the heart tube [44,45,46].

There is still no consensus on the boundaries and fate of the heart fields; however, cell tracking studies on chick embryos have generated novel information on this aspect. At the beginning of this century, it was concluded that the splanchnic mesoderm of the SHF cranial to the straight heart tube gives rise to the embryonic outflow tract, which includes the segments called the conus and truncus (some authors also include the aortic sac). The cranial SHF would eventually form the ventricular outflow tracts and arterial trunks. However, recent research contradicts this idea. Some important findings have been revealed by selective labeling of the conus boundaries and its walls, together with tracking of labeled populations up to mature heart and histological analysis. It was found that as the conus wall opens, the myocardium gradually redistributes to the right ventricle. As a result, the proximal region of the conus gives rise to the right ventricle (Figure 4B–F), whereas the distal region participates in the development of the right ventricular outflow tract (Figure 4D–F). It was also observed that the process of opening the dextrodorsal wall is independent of apoptosis [16,47]. Regarding the origin of the left ventricular outflow tract, in vivo labeling studies have revealed that the aortic vestibule is formed from the ventro-superior cushion of the atrioventricular canal [47]. On the contrary, the caudal or posterior population of the SHF forms the atria and sinoatrial node (Figure 4B–F). The FHF integrates the straight cardiac tube and is composed of caudal and cephalic segments. The caudal segment contributes to the left ventricle and parts of the atrioventricular canal. The cephalic segment is involved in the formation of the interventricular septum instead of giving rise to the right ventricle, as believed previously (Figure 4A–F) [16]. These new findings demonstrate that the gradual and sequential integration of the different primordial cardiac segments from the SHF during the heart tube’s twisting and folding processes must be reconsidered.

## 5. Twisting, Folding, and Rotation of the Heart Tube

This process is crucial for the formation of the four heart chambers as well as their correct interaction and connection. As the linear heart tube grows, the cardiac looping process begins. It can be divided into four stages: (1) formation of a C-shaped loop; (2) transformation into an S-shaped or D-shaped loop (CS-10, days 21–22); (3) advanced loop stage (~day 25); and late loop stage (~day 30). Based on recent labeling experiments for determining the boundaries of the straight heart tube and tracing the fate of the labeled cells until they become a mature heart, a new model for the segmental patterning of the heart tube has been proposed. This model differs from the currently accepted model in terms of the incorporation of the FHF and SHF and the processes of twisting and folding the heart tube [16]. It states that the straight heart tube is initially formed by two segments. The anterior or cephalic segment is involved in the development of the interventricular septum (Compare Figure 4A with Figure 4F) but not in the formation of the right ventricle, as previously proposed [34]. The posterior bifurcated segment is involved in the development of the left ventricle and the proximal part of the atrioventricular canal (Figure 4A,F) [16,48] but not in the formation of the atria. As the splanchnic mesoderm of the SHF is recruited, the embryo continues to bend cranially and cervically, and new segments emerge from the tubular heart [49]. During C-loop formation (Figure 4B,G), the ventral side of the primary heart tube becomes the outer curvature of the heart loop, whereas the dorsal side becomes the inner curvature. The initial development of the proximal portion of the conus can be observed at the cephalic border of the C-loop. The conus later develops into the right ventricle [47,48] (Figure 4B,F). Meanwhile, at the caudal end of the C-loop are the precursors of the tubular-shaped atrioventricular canal and the primitive atria with a still bifurcated appearance (Figure 4B,G). In the S-loop (days 21–22 in humans), the proximal part of the conus (right ventricular primordium) continues to be incorporated into the cephalic border of the loop; the left ventricular primordium along with the precursors of the interventricular septum begin to descend; and the primitive atria are more conspicuous and begin to ascend (Figure 4C,G). Subsequently, the distal end of the conus can be distinguished in the advanced loop (~day 25) [16]. The incipient right ventricular primordium, derived from the proximal end of the conus, and the well-developed left ventricular primordium, which are separated by the precursors of the interventricular septum, occupy a caudal position, whereas the primitive atria present a dorsocephalic arrangement (Figure 4D). At this stage, the distal segment of the conus, which is more developed and connected to the right trabecular region, rotates to the left and gets positioned ventrally (Figure 4D–F,G). The primitive inflow tract (atrioventricular canal), also a precursor of the left ventricular outflow tract or aortic vestibule [47,50,51,52], is connected to the trabecular region of the left ventricle and is slightly shifted to the right (Figure 4D). At the late loop stage, the ventro-superior and dorso-inferior cushions of the atrioventricular canal and trabeculae in the apical wall of the ventricles are observed (~day 30), and the truncus with its myocardial walls, the precursor of the aortic and pulmonary valves, and the insertion annulus appear [53] (Figure 4E,G). Based on the labeling and tracing experiments mentioned earlier and the fact that the aortic sac shows a vascular composition from early development and is divided by neural crest cells into the pulmonary and aortic conduits, it has been assumed that the aortic sac is the precursor of the aortic and pulmonary arteries [53,54].

Shortly thereafter, the embryonic outflow tract and the atrioventricular canal move closer to the midline through a process called convergence, which aligns these structures. In the final step, the truncus is divided into systemic (aorta) and pulmonary trunks by a process known as wedging, which describes the counterclockwise rotation of the outflow tract with the movement of the future aortic valve position behind the pulmonary trunk. Taken together, all of the changes in the position of the embryonic heart structures determine a new spatial orientation of the primitive heart segments, ensuring the proper development of the cardiac septa and valves as well as the correct connection and interaction of the heart chambers. Animal models and human clinical trials show that errors in the twisting, folding, or rotation of the heart tube are associated with complex CHDs [17] (Table 1). However, there is no agreement on the cellular mechanisms, morphogenetic processes, and molecular networks involved.

## 6. Twisting, Folding, and Rotation Mechanisms of the Heart Tube

It has been suggested that the rightward twisting of the heart tube to form the C-loop depends on an increased supply of splanchnic mesoderm cells on the left side. In line with this theory, there have been reports on the differential expression of genes coding for proteins that promote, directly or indirectly, changes in the cytoskeletal organization and extracellular matrix. Among these are the growth factors Nodal and Hedgehog and the transcription factors Pitx2 and Nkx 2.5, which act either individually or together with Mef2c, Hand1, and/or Hand2. In addition, considering that straight heart tubes explanted and cultured in vitro demonstrated the innate ability to undergo tubulation and almost always twist to the right [16,55], it has been suggested that heart tube twisting is an intrinsic process in which Tbx5 plays a major role [56].

## 7. Establishment of the Left–Right Axis

**Figure 5:**
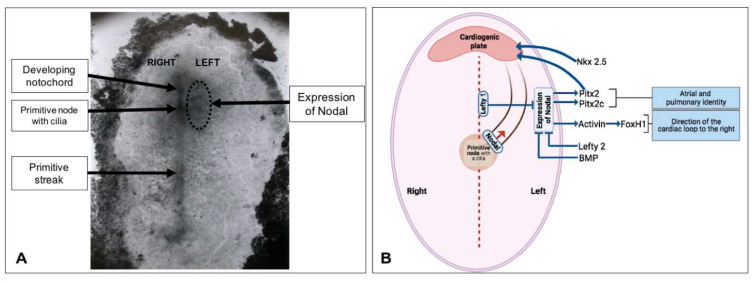
Model for Nodal signalling in the left-lateral plate mesoderm and activation Pitx2 in the heart morphogenesis in normal embryos. In the initial event, that translates the motion of primitive nodal cilia (Hensen) toward the left side of the chicken embryo, which regulates the expression of Foxh1 and Pitx2c through the receptor of the activin signaling pathway. Foxh1 determines the rightward direction of the heart loop and the ventricular topology. Pitx2c expression determines the atrial identity. (**A**) Real embryo. (**B**) Representation of the possible flow of nodal from the primitive node and the interacting genes. (The PS is exemplified in the central dotted line). Based on Chen CM, Norris D, Bhattacharya S. Transcriptional control of left-right patterning in cardiac development [57].

In humans, the position of the visceral and abdominal organs is asymmetrical in relation to the two main body axes. This arrangement, called *situs solitus*, is rarely modified. Only about 1 in 10,000 humans has internal organ arrangements that are mirror images of the normal organ arrangements, a condition known as *situs inversus*. At the end of the 3rd week, during the specialization period of cardiac cells, Hensen’s node gives rise to the development and lateralization of the right and left structures. One of the first signs of asymmetry in the embryo becomes apparent when the straight heart tube begins to bend to the right (CS-9). The molecular mechanisms that facilitate this break in symmetry are related to the right–left motion of primitive nodal cilia, which creates a leftward flow of the intracellular fluid. This, in turn, activates the expression of Nodal, a member of the TGF-β family of growth factors, on the left side. The propagation of this signal to the right side is inhibited by the expression of the Nodal inhibitor Lefty1 in the midline [57,58]. The Nodal signaling pathway activates the activin signaling pathway, leading to the expression of Pitx2c, an important regulator of left-sided signaling in the body (Figure 5). The alteration of the right–left flow due to changes in the function of the cilia results in randomization of the left–right pattern. The disruption of Nodal signaling results in the loss of left-sided signaling and, consequently, right isomerism. Interestingly, heart looping appears to be independent of Pitx2c. The PITX2c mutants show right isomerism and, thus, two morphologically right atria; however, the heart loop remains unaffected. This indicates that in addition to the Pitx2c pathway, other signaling pathways are involved in heart tube twisting [23]. A recent study proposed that the intrinsic chirality of the heart tube cells drives the rightward twisting of the heart tube [56]. It is worth mentioning that the genetic and experimental data clearly support the cilia-mediated flow model in fish, amphibians, and some mammals such as mice, rabbits, and humans; however, nodal cilia have not been found in chick embryos and are apparently absent in pigs [58].

## 8. Atria, Loop, and Visceroatrial Situs

The ventricles develop in series and from different heart fields. The right ventricle (derived from the SHF) is formed above the left ventricle (derived from the FHF). Consequently, there is no ventricular isomerism. In contrast, the atria develop in a symmetrical bilateral fashion and are influenced from the beginning by left–right signaling pathways, developing differently based on the signals received. Likewise, the left–right pattern drives the connection of the outflow tracts with their corresponding ventricles and the development of the arteries in the pharyngeal arches. The connections of the different veins to the atria and the development of the veins themselves, e.g., the left superior vena cava or the azygos system, are also influenced by the left–right signaling pathways. Thus, the topology and anatomical characteristics of each of the atria, including the atrial appendages and their relationship with the suprahepatic segment of the inferior vena cava, make the hepato–cavo–atrial complex the appropriate anatomical element for the diagnosis of visceral situs [59].

## 9. Start of Septation

Cardiac jelly, present between the myocardium and endocardium from the beginning of heart development, thins in the ventricular region, where the trabeculae will later develop. In contrast, cardiac jelly begins to accumulate in the embryonic inflow (atrioventricular canal) and outflow (conus and truncus) tracts and is later invaded by mesenchymal cells. The latter results from the transformation of the endocardium into mesenchyme to form the cushions of the atrioventricular canal and the ridges of the truncus and conus. The mesenchymal tissue in these regions prevents regurgitation and ensures directional blood flow in the embryonic heart. Mesenchymal tissue formation is triggered by myocardium-derived BMP signals that activate the Notch and TGF-β signaling pathways [60]. The endocardium-derived mesenchyme in the cushion later gives rise to the definitive heart valves. At this stage, the cardiac neural crest cells that enter the arterial pole of the heart contribute to the mesenchyme in the cushions of the distal outflow tract or truncus [61]; however, the extent of their contribution to the mesenchyme in the cushions of the atrioventricular canal remains unknown.

**Figure 6:**
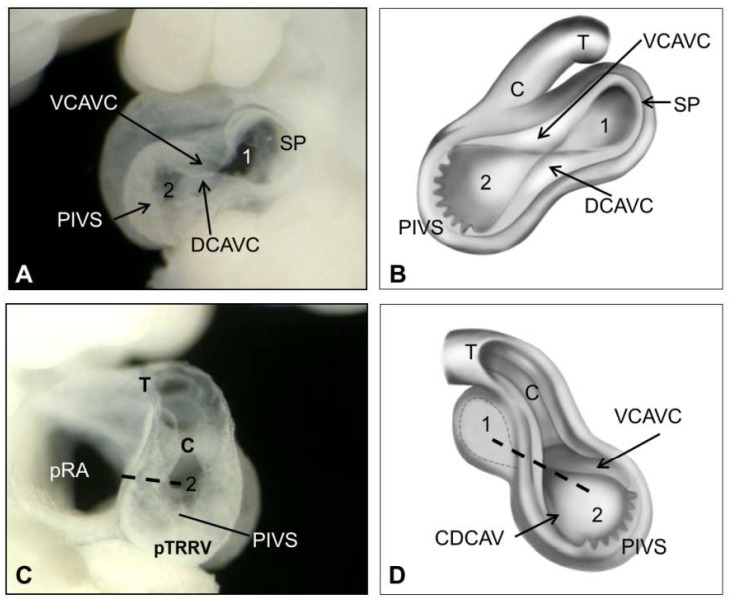
Onset of cardiac septation in a rat embryo of an age equivalent to 28–32 days in humans. (**A**,**B**) Sagittal view of the left cavities. Note the mask-shaped primitive cardiac septum composed of the septum primum (SP), the primitive interventricular septum (PIVS), and the ventral (VCAVC) and dorsal (DCAVC) cushions of the atrioventricular canal. Note the foramen primum (1) and the primitive interventricular foramen (2). The structures are in the same plane without demarcation of limits. (**C**,**D**) Sagittal view of the right cavities showing the connection between the primitive right atrium (PRA) and its ventricle (black dotted line). Note that although the four-chambered heart has begun to establish, a single conus (**C**) emerges from the developing right ventricle (PTRRV). Abbreviation: T: truncus.

The first anatomical sign of septation is the primitive cardiac septum, which consists of three elements (29 ± 1 dpc, Figure 6): (1) The *septum primum* with the *foramen primum* at the atrial level; (2) The ventral (superior) and dorsal (inferior) cushions of the atrioventricular canal that partially divide the primitive inflow tract (common atrioventricular opening) into a very narrow opening on the right (connecting the embryonic right atrium with its corresponding ventricle) and a wider opening on the left (connecting the left atrium and left ventricle); and (3) A group of incipient trabeculae lining the primary interventricular *foramen* in the apical ventricular region. At this stage, a four-chambered heart starts to form, the right ventricle already has an incipient inflow chamber, and the left ventricle still lacks an outflow chamber [62].

## 10. Frontier Techniques for the Study of Heart Development

Classic embryology techniques are fundamental in the study of organ development, including that of the heart. Furthermore, in vitro models have currently been designed to study embryonic development. Breakthroughs in the study of stem cells have enabled researchers to design ex vivo models that mimic the embryonic microenvironment during heart development. The two main models are gastruloids and cardiac organoids.

Van den Brink and Van Oudenaarden define gastruloids as “embryonic stem cell aggregates that recapitulate the gastrulation stage in vitro” [63]. Furthermore, organoids are miniature, three-dimensional structures that originate from pluripotent stem cells and recapitulate cellular heterogeneity, structure, and organ functions [64]. The organoid and gastruloid models, along with tools for expression analysis, including single-cell RNAseq (scRNAseq), are an important advancement for understanding processes that direct the development of different organs, including the heart, at the cellular and molecular levels.

Moris et al. reported that when human embryonic cells were stimulated with Chir9901, the WNT pathway was activated, resulting in the formation of aggregates (gastruloides) that expressed specific markers of the mesoderm, endoderm, and neurectoderm in specific territories (i.e., separate layers). Gene expression analysis using scRNAseq technology revealed the presence of the first and second cardiac field markers [65].

Minn et al., also established a protocol for human gastruloids using human embryonic cells and observed that when the cells were stimulated with BMP4 (an important regulator of embryonic development), they could differentiate into the three germ layers. Moreover, scRNAseq technology confirmed the presence of different types of cardiogenic cells [66].

Olmsted and Paluh designed a gastruloid model displaying neurocardiac lineages. Furthermore, this model recapitulates numerous features involved in cardiac development. This group had previously generated gastruloids with central and peripheral neurons [67,68] and tested whether these gastruloids could model cardiogenesis; the exposure was modified according to growth factors during the early stages of formation. This model is known as EMLOCs, and it was generated using the hiPSC H3.1.1 line. Reportedly, through scRNAseq technology, the mesoderm not only gives rise to cardiomyocytes but also contributes to the formation of the epicardium, endocardium, connective tissue, outflow tract, valves, and conduction apparatus. The cells express cardiac neural crest biomarkers in addition to calcium signaling and spontaneous contractility [69].

An in vitro model based on the culture of embryonic stem cells was designed by the Zernicka-Goetz group, which captures the interactions between embryonic and extraembryonic tissues. This model recreates events of embryonic development such as gastrulation, formation of the anterior–posterior axis of the brain, and a contractile structure similar to a heart. Additionally, it mimics the development of extraembryonic tissues, including the yolk sac and the chorion [70].

The in vitro generation of cardiac organoids from induced stem cells (including human stem cells) has become an important tool in the study of cardiac tissue development. Cardiomyocytes that differentiate into organoids show greater signs of maturation, unlike gastruloids [71]. It is still difficult to ensure adequate microarchitecture of the tissues as well as the spatial orientation of the different types of cells in the culture of organoids, aside from ensuring adequate nutrient supply in all areas and adequate elimination of cellular metabolites [72].

Fumitoshi Ishino’s group was possibly the first to report the generation of cardiac organoids from mouse embryonic cells, with structures similar to those of the atria and ventricles. To generate these organoids, the investigators cultivated the cells in an extracellular matrix enriched with a laminin–entactin complex under serum-free conditions and with fibroblast-derived growth factor as a stimulant. They obtained organoids with different cell types, such as cardiomyocytes, cardiac endothelial cells, smooth muscle cells, and cardiac neurons [73].

Drakhlis et al., described a method for recapitulating the first steps of human cardiogenesis in vitro. For this purpose, organoids called heart-forming organoids were generated from human pluripotent cells. In this methodology, the strategies of encapsulation with matrigel and modulation of the WNT pathway are combined. These organoids are composed of a myocardial layer covered with endocardial cells and surrounded by cells of the anterior and posterior foregut endoderms, where a vascular network can also be seen [74].

One of the main differences between gastruloids and cardiac organoids is in the main signaling pathways that regulate their development. Furthermore, cardiac organoids can be cultured for long periods. Gastruloids are an important tool for studying the embryonic development of the heart during gastrulation. The information provided in this article on the studies of in vivo labeling and cell tracking will support these frontier technologies. Their results can be interpreted in the context of the progressive anatomical and histological changes in the embryonic heart, which together provide a better understanding of the origins of congenital heart disease.

## 11. Conclusions

The new knowledge about the origin of the left ventricle from the FHF and the right ventricle from the SHF should be considered while planning surgical strategies for the correction of pathologies involving the ventricular cavities of the heart. The contribution of the SHF originating outside the primitive heart tube may explain the co-occurrence of CHDs and extracardiac anomalies in patients with genetic syndromes. In addition, the possible invasion of neural crest cells into the mesenchyme in the atrioventricular canal cushions and their possible involvement in the development of the left ventricular outflow tract should be investigated in the future to better understand the origin of CHDs affecting this region of the heart in cardiopathies.

The study results demonstrated that new frontier technologies should consider a new vision of cardiac development based on in vivo labeling and cell tracking for a better understanding of cardiac morphogenesis in health and disease conditions.

## Figures and Tables

**Table 1:** Heart field and contribution of neural crest cells to the structures of the human heart [16,17] and implicated congenital heart diseases [18,19,20].

Embryonic Origin	Contribution to Heart Morphogenesis	Cell Lineages Derived	Mature Structures Formed	Congenital Heart Disease
First heart field	It forms the cardiac crescent (days 18–20), which gives rise to the straight heart tube (day 19)	Endocardium and myocardium	Trabeculated region of the left ventricle Some atrioventricular populations Involved in the development of the interventricular septum	Left ventricular hypoplasia Univentricular heart Double outlet of the left ventricle Ventricular septal defect
Second heart field	It is incorporated into the heart during the twisting and folding processes (days 22–23)	Endocardium, myocardium, cushion mesenchyme in the atrioventricular canal, and ridges of conus and truncus	Trabeculated region of the right ventricle Ventricular inflow and outflow tracts Aortic and pulmonary valves and insertion annulus Atria and sinoatrial node	Double outlet of the right ventricle Persistent atrioventricular canal Pulmonary atresia Tricuspid atresia
Cardiac neural crest cells	They delaminate from the cranial neural crest and migrate into the 3rd, 4th, and 6th pharyngeal arches; during recruitment from the SHF to the heart tube, they travel to the embryonic outflow tract until they reach the endocardial cushions and ridges	Fibroblasts, endothelium, smooth muscle cells, cardiomyocytes, neurons, and glial cells	Heart valves Great arteries Conduction system Cardiac ganglia	Truncus arteriosus Aortopulmonary window Transposition of the greater arteries Congenital long QT syndrome

## Data Availability

Not applicable.

## Abbreviations

AP	Angiogenic plexuses
AVC	Atrioventricular canal
BMP	Bone morphogenetic protein
C	Conus
CHD	Congenital heart defects
CP	Cardiogenic plate
DCAVC	Dorsal cushions of the atrioventricular canal
EnT	Endocardial tube
FG	Foregut
FHF	First heart field
IVS	Interventricular septum
LA	Left atrium
LCA	Left cardiogenic area
LV	Left ventricle
OPN	Oropharyngeal membrane
pAVC	Primordium of the atrioventricular canal
PC	Pericardial cavity
PIVS	Primitive interventricular septum
PLA	Primitive left atrium
pLV	Primordium of the left ventricle
pRV	Primordium of the right ventricle,
PN	Primitive node
PRA	Primitive right atrium
PS	Primitive streak
RCA	Right cardiogenic area
RA	Right atrium,
RV	Right ventricle
RVOT	Right ventricle outflow tract
SHF	Second heart field
SHT	Straight heart tube
SP	Septum primum
VCAVC	Atrioventricular canal ventral cushions
T	Truncus.
Sig	Sigmoid valves
scRNAseq	Single-cell RNAseq
